# Genome-wide identification and expression analysis of the KNOX family and its diverse roles in response to growth and abiotic tolerance in sweet potato and its two diploid relatives

**DOI:** 10.1186/s12864-024-10470-4

**Published:** 2024-06-06

**Authors:** Li-Cong Jia, Zi-Tong Yang, Li-Li Shang, Shao-Zhen He, Huan Zhang, Xu Li, Guo-Sheng Xin

**Affiliations:** 1 Institute of Grain and Oil Crops, Yantai Academy of Agricultural Sciences, Yantai, 265500 China; 2 Key Laboratory of Sweet Potato Biology and Biotechnology, Ministry of Agriculture and Rural Affairs/Beijing Key Laboratory of Crop Genetic Improvement/Laboratory of Crop Heterosis & Utilization and Joint Laboratory for International Cooperation in Crop Molecular Breeding, Ministry of Education, College of Agronomy & Biotechnology, China Agricultural University, Beijing, 100193 China; 3 Sanya Institute of China Agricultural University, Hainan, 572025 China

**Keywords:** Sweet potato, *I. trifida*, *I. triloba*, *KNOX*, Tissue-specific expression, Hormone treatment, Abiotic stress

## Abstract

*KNOXs*, a type of homeobox genes that encode atypical homeobox proteins, play an essential role in the regulation of growth and development, hormonal response, and abiotic stress in plants. However, the *KNOX* gene family has not been explored in sweet potato. In this study, through sequence alignment, genomic structure analysis, and phylogenetic characterization, 17, 12 and 11 *KNOX*s in sweet potato (*I. batatas*, 2n = 6x = 90) and its two diploid relatives *I. trifida* (2n = 2x = 30) and *I. triloba* (2n = 2x = 30) were identified. The protein physicochemical properties, chromosome localization, phylogenetic relationships, gene structure, protein interaction network, *cis*-elements of promoters, tissue-specific expression and expression patterns under hormone treatment and abiotic stresses of these 40 *KNOX* genes were systematically studied. *IbKNOX4, -5*, and *− 6* were highly expressed in the leaves of the high-yield varieties Longshu9 and Xushu18. *IbKNOX3* and *IbKNOX8* in Class I were upregulated in initial storage roots compared to fibrous roots. *IbKNOXs* in Class M were specifically expressed in the stem tip and hardly expressed in other tissues. Moreover, *IbKNOX2* and *− 6*, and their homologous genes were induced by PEG/mannitol and NaCl treatments. The results showed that *KNOXs* were involved in regulating growth and development, hormone crosstalk and abiotic stress responses between sweet potato and its two diploid relatives. This study provides a comparison of these *KNOX* genes in sweet potato and its two diploid relatives and a theoretical basis for functional studies.

**Supplementary Information:**

The online version contains supplementary material available at 10.1186/s12864-024-10470-4.

## Background

The homeobox (HB) genes encode transcription factors (TFs) that contain a homeobox domain, also known as a homeodomain (HD), which play an important role in plant growth and development [1]. The HB genes have been categorized into 14 classes based on their structural characteristics, including HD-ZIP I, HD-ZIP II, HD-ZIP III, HD-ZIP IV, PLINC, WOX, DDT, PHD, NDX, LD, PINTOX, SAWADEE, BEL, and KNOX [2]. The KNOX (KNOTTED1-like homeobox) gene family plays an important regulatory role in plant morphogenesis, pattern formation, and other processes. With the continuous development and progress of plant genomics, the first *KNOX* gene was discovered in maize [3]. Genome-wide analysis led to the identification of *KNOX* genes in various plants, such as *Arabidopsis* [4], rice [5], maize [6], wheat [7], cotton [8], tobacco [9], tomato [2], soybean [10], radish [11], potato [12], cassava [13] and *Phyllostachys edulis* [14]. KNOX proteins generally contain four characteristic domains: KNOX1, KNOX2, ELK and Homeobox-KN [4]. The KNOX1 and KNOX2 domains of the N-terminus are connected by a poorly conserved splice sequence to form the MEINOX domain, which is followed by the ELK domain and the Homeobox-KN domain [15]. Based on their structural characteristics, phylogenetic relationships and expression patterns, KNOXs can be divided into three Classes: Class I, Class II and Class M [16].

In *Arabidopsis*, Class I KNOX genes are mainly expressed in the apical meristem and are involved in the regulation of plant hormones and plant multiorgan morphogenesis [17–19]. In tobacco, *NtKNATM1* might be positively regulated by auxin and participate in the development of apical and lateral tissues [20]. *TaKNOX1s* in wheat was a positive regulator of wheat grain size and grain weight and was also related to the regulation of wheat plant type [21]. The rice KNOX II protein HOS59 negatively regulated rice glial cell length, rice grain size, and plant structure [22]. Moreover, the *KNOX* gene family plays an important role in the response to abiotic stress [7, 8]. *TaKNOX11-A* transgenic plants exhibited enhanced tolerance to drought and salt stress [23]. The Class *KNOX* I gene *PagKNAT2/6b* mediated changes in plant architecture in response to drought by downregulating *GA20ox1* in *Populus alba* × *P. glandulosa* [24]. Overexpression of *STM* in *Arabidopsis* resulted in enhanced tolerance to drought stress [25]. In sweet potato, *KNOX* I genes had been reported to be involved in the development of sweet potato storage roots and regulate the level of cytokinin in storage roots [26]. *Ibkn1- Ibkn3* were highly expressed in storage roots than in fibrous roots [27]. However, the mechanism of *Ibkn1- Ibkn3* and the expression patterns of other *KNOXs* in sweet potato are still unknown.

Sweet potato (*Ipomoea batatas* (L.) Lam, 2n = B_1_B_1_B_2_B_2_B_2_B_2_ = 6x = 90) is an important food crop, as well as a high-quality raw material for feed and industry [28]. Due to its robust adaptability, extensive planting range, high yield and high nutritional value, sweet potato has a long history of cultivation in China [29]. However, with limited land availability, sweet potato cultivation constitutes merely approximately 3% of the total cultivated land area, significantly less than wheat, corn, and rice [30]. Soil salinization caused by industrial pollution and abuse of fertilizers and pesticides [30], as well as extreme weather, have also impacted the yield and quality of sweet potato [31]. With the completion of genome sequencing and assembly of hexaploid sweet potato Taizhong 6 and its two diploid relatives, *Ipomoea trifida*, NCNSP0306 (2n = 2x = 30) and *Ipomoea triloba*, NCNSP0323 (2n = 2x = 30) [32, 33], it is feasible to analyze and identify essential gene families at the whole genome level of sweet potato to improve the yield and quality of sweet potato.

In this study, the *KNOX* gene family members of sweet potato and its two diploid relatives were identified. They were classified into three Classes. Through comprehensive analysis of protein physicochemical properties, chromosome localization, phylogenetic relationships, gene structure, *cis*-elements of promoters, protein interaction networks and expression patterns in different tissues, hormones, and abiotic stresses by RNA-seq, we obtained a preliminary understanding of the evolution and function of *KNOXs*, which provided a theoretical basis for enhancing stress resistance, yield and quality in sweet potato.

## Materials and methods

### Plant materials

Sweet potato (*I. batatas*) and its two diploid relatives (*I. trifida* and *I. triloba*) were used in this study. The drought/salt-sensitive sweet potato variety Lizixiang (lzx), the salt-tolerent sweet potato line ND98 [34], the drought-tolerant sweet potato line Xushu55-2 (Xu55-2) [35] and two diploid relatives were used to analysis the expression pattern of *KNOXs* in abiotic stresses. Two diploid relatives and the sweet potato cultivar Xushu22 (Xu22) [36], Longshu9 with high yield and early maturity (Long9) [37], Xushu18 with high yield (Xu18) [38] were used to analysis the expression pattern of *KNOXs* in different tissues and periods.

### Identification of *KNOXs*

The whole-genome sequences of *I. batatas*, *I. trifida*, and *I. triloba* were downloaded from the *Ipomoea* Genome Hub (https://ipomoea-genome.org/) and Sweetpotato Genomics Resource (http://sweetpotato.plantbiology.msu.edu/). To ensure the accuracy of the identification results, we integrated three screening methods. First, we used all *AtKNATs* from the *Arabidopsis* genome database (https://www.arabidopsis.org/) as queries to predict *KNOXs* through the BLAST algorithm (BLASTP, E value ≤ 1 × 10^− 5^) [16]. Next, potential *KNOXs* were identified by HMMER 3.0 software through hidden Markov Model profiles (hmmsearch, E value ≤ 1 × 10^− 5^) of the KNOX1 domain (pfam03790) and KNOX2 domain (pfam03791), which were extracted from the Pfam databases (http://pfam.xfam.org/) [39]. Finally, all putative *KNOXs* were verified using CD-search (https://www.ncbi.nlm.nih.gov/Structure/cdd/wrpsb.cgi) [40–42].

### Protein property prediction of KNOXs

The molecular weight, theoretical isoelectric point, instability index and hydrophilicity of IbKNOX proteins were calculated by ExPASy (https://www.expasy.org/) [43], and the subcellular localization was predicted by PSORT (https://wolfpsort.hgc.jp/).

### Chromosomal distribution of *KNOXs*

The positional information on chromosomes of *KNOXs* in sweet potato and their two diploid relatives were obtained from *Ipomoea* Genome Hub (https://ipomoea-genome.org/) and Sweetpotato Genomics Resource (http://sweetpotato.plantbiology.msu.edu/). The visualization was generated by TBtools software (v.1.098775) [44].

### Phylogenetic analysis of KNOXs

First, MAFFT version 7 (https://mafft.cbrc.jp/alignment/server/) [45, 46] was used to align the protein sequences of *Arabidopsis*, *I. batatas*, *I. trifida* and *I. triloba*. Then, we selected the maximum likelihood method, AIC model and a bootstrap value of 500 to construct a phylogenetic tree by PhyML 3.0 (http://www.atgc-montpellier.fr/phyml/) [47]. The evolutionary trees of sweet potato and their two diploid relatives were also constructed in this way. Finally, the phylogenetic tree was visualized on Evolview (http://www.evolgenius.info/evolview/) [48–50].

### Conserved domains and exon‒intron structure

The structural domain information of each protein was obtained from NCBI-CDD (https://www.ncbi.nlm.nih.gov/Structure/cdd/wrpsb.cgi) [40–42], and the exon‒intron structures of *KNOX* genes were obtained by GSDS 2.0 (http://gsds.gao-lab.org/) [51]. They were visualized by TBtools software (v.1.098775) [44].

### Promoter analysis of *KNOXs*

The *cis*-elements of the approximately 2000 bp promoter region upstream of the *KNOX* gene in sweet potato were predicted by PlantCARE (https://bioinformatics.psb.ugent.be/webtools/plantcare/html/) [52].

### Protein interaction network of KNOXs

The KNOX protein interaction network of sweet potato was predicted based on homologous proteins from *Arabidopsis* with a confidence level of 0.04 by using STRING (https://cn.string-db.org/), and the network map was visualized by using Cytoscape software [53].

### Transcriptome analysis of *KNOXs*

The RNA-seq data of *IbKNOXs* in Long 9 and Xu18 were unpublished. The RNA-seq data of *IbKNOXs* in Xu55-2, ND98 and Xu22 were obtained from NCBI Sequence Read Archive (SRA, http://www.ncbi.nlm.nih.gov/Traces/sra) with accession number SRP092215 [34], PRJNA999504 [35] and SAMN10755180-SAMN10755194 [36], respectively. The RNA-seq data of *ItfKNOXs* and *ItbKNOXs* in *I. trifida* and *I. triloba* were downloaded from the Sweetpotato Genomics Resource (http://sweetpotato.plantbiology.msu.edu/). The expression levels of *KNOXs* were calculated as fragments per kilobase of exon per million fragments mapped (FPKM). The expression level was shown as the log_2_(FPKM), and heatmaps were constructed by TBtools software (v.1.098775) [44].

### Expression analysis of *IbKNOXs*

Total RNA was extracted from the leaves of 4-week-old in vitro-grown Xu18 plants treated with 20% PEG6000 and ND98 plants treated with 200 mM NaCl in half-Hoagland solution. Experiments were conducted with three biological replicates, each with three plants. Transcript abundances were determined using reverse-transcription quantitative polymerase chain reaction (ZF502; ZOMANBIO, Beijing, China). The expression of *IbKNOXs* were measured and the sweet potato *β-actin* (AY905538) gene was used as the internal control (Table S1). Gene expression was quantified using the comparative C_T_ method [54].

## Results

### Identification and characteristics of *KNOXs* in sweet potato and its two diploid relatives

In this study, BLASTP, hmmersearch and CD-search were employed to screen *KNOXs* of sweet potato and its two diploid relatives. Based on the screening results, a total of 40 *KNOX* genes were identified, including 17 in *I. batatas*, 12 in *I. trifida*, and 11 in *I. triloba* (named after “*Ib*”, “*Itf*”, and “*Itb*”). According to their chromosome positions, these genes were named *IbKNOX1 ~ IbKNOX17*, *ItfKNOX1 ~ ItfKNOX12*, and *ItbKNOX1 ~ ItbKNOX11*. The sequence attributes of *IbKNOXs* and their physicochemical properties were analyzed (Table 1). The genome length of *IbKNOXs* ranged from 1903 bp (*IbKNOX17*) to 8508 bp (*IbKNOX2*), while the length of CDS varied from 441 bp (*IbKNOX1, IbKNOX12*) to 1614 bp (*IbKNOX15*). The amino acid length of IbKNOXs ranged from 146 aa (IbKNOX1, IbKNOX12) to 537 aa (IbKNOX15). The molecular weight ranged from 16.623 kDa (IbKNOX1, IbKNOX12) to 59.589 kDa (IbKNOX15). The isoelectric point distribution is between 4.26 (IbKNOX13) and 9.98 (IbKNOX17), with only IbKNOX17 being an alkaline protein with an isoelectric point exceeding 7, while others were acidic proteins. Except for IbKNOX3 and IbKNOX17, the instability index of the other IbKNOXs was greater than 41, indicating that they are unstable. The GRAVY scores of all IbKNOXs were negative, suggesting that they were hydrophilic proteins, with IbKNOX9 being the most hydrophilic and IbKNOX17 the least hydrophilic. The subcellular localization prediction revealed that all IbKNOXs might be localized in the nucleus.

**Fig. 1:**
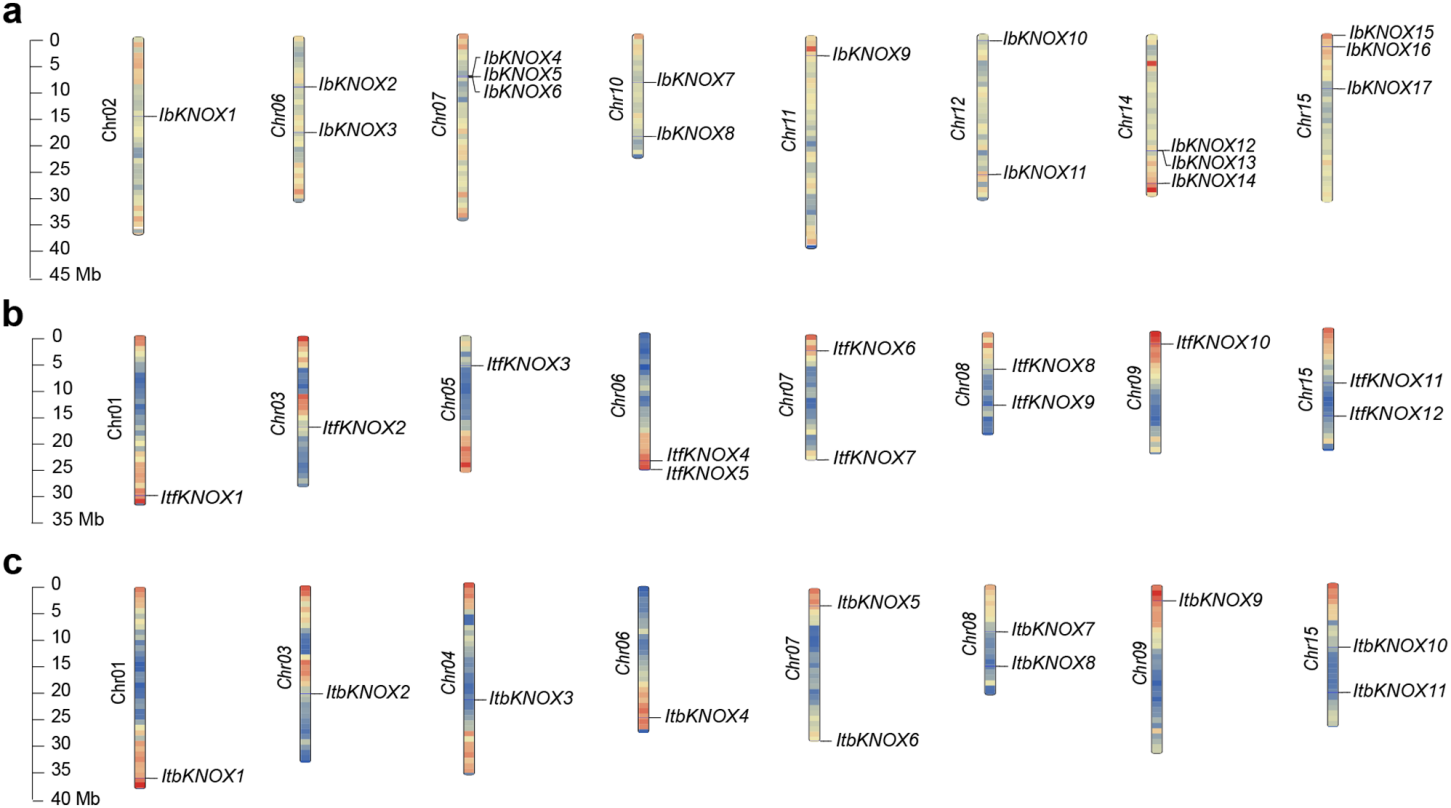
Chromosomal localization and distribution of *IbKNOXs* (**a**), *ItfKNOXs* (**b**) and *ItbKNOXs* (**c**). The bars represented chromosomes, the chromosome numbers were displayed on the left side, and the gene names were displayed on the right side

The *KNOXs* of *I. batatas*, *I. trifida*, and *I. triloba* were distributed across eight chromosomes (Fig. 1). In *I. batatas*, three *IbKNOXs* were detected on Chr07, Chr14 and Chr15, two on Chr06, Chr10 and Chr12, and one on Chr02 and Chr11. No genes were detected on Chr01, Chr03, Chr04, Chr05, Chr08, Chr09 and Chr13 (Fig. 1a). By comparing the chromosomal localization of *KNOXs* in *I. trifida* and *I. triloba*, we observed a slight difference, where there is one more gene (*ItfKNOX5*) on Chr06 of *I. trifida* than *I. triloba* (Fig. 1b and c). The remaining *KNOXs* on other chromosomes of the two diploid relatives were distributed similarly, with one gene on Chr01, Chr03, Chr05/04, and Chr09 and two on Chr07, Chr08, and Chr15 (Fig. 1b and c). The distribution of *KNOX* genes in sweet potato and its two diploid relatives differed significantly, indicating that *KNOX* genes in sweet potato had undergone some variation and loss in the process of evolution.

**Table 1:** Characterization of *IbKNOXs* in sweet potato

Gene name	Gene ID	Genomic length (bp)	CDS length (bp)	Protein size (aa)	MW (kDa)	pI	Instability index	Gravy	Subcellular locations
*IbKNOX1*	g6362	2299	441	146	16.623	4.39	53.97	-0.697	Nucleus
*IbKNOX2*	g22159	8508	1341	446	48.835	5.34	56.47	-0.555	Nucleus
*IbKNOX3*	g23341	2532	621	206	22.659	5.12	39.55	-0.473	Nucleus
*IbKNOX4*	g26410	4395	1218	405	45.287	5.8	58.6	-0.809	Nucleus
*IbKNOX5*	g26425	4603	1158	385	43.189	5.84	58.16	-0.726	Nucleus
*IbKNOX6*	g26440	4463	1194	397	44.627	5.9	59.11	-0.87	Nucleus
*IbKNOX7*	g39428	4799	912	303	33.799	6.07	64.3	-0.58	Nucleus
*IbKNOX8*	g40812	4271	591	196	21.590	4.4	42.79	-0.356	Nucleus
*IbKNOX9*	g41979	4364	1191	396	44.496	6.29	41.59	-0.917	Nucleus
*IbKNOX10*	g47080	2815	1161	386	43.502	6.21	64.46	-0.817	Nucleus
*IbKNOX11*	g50432	3944	771	256	28.601	5.04	41.73	-0.4	Nucleus
*IbKNOX12*	g58404	3077	441	146	16.623	4.39	53.97	-0.697	Nucleus
*IbKNOX13*	g58406	2869	456	151	17.196	4.26	52.52	-0.736	Nucleus
*IbKNOX14*	g59362	4190	966	321	35.759	5.6	46.72	-0.645	Nucleus
*IbKNOX15*	g59905	8210	1614	537	59.589	6.22	46.02	-0.448	Nucleus
*IbKNOX16*	g60276	3865	1074	357	40.200	5.09	51.73	-0.736	Nucleus
*IbKNOX17*	g61435	1903	648	215	23.861	9.98	17.29	-0.236	Nucleus

*CDS* coding sequence, *MW* molecular weight, *pI* isoelectric point

### Phylogenetic relationship of KNOXs in sweet potato and its two diploid relatives

**Fig. 2:**
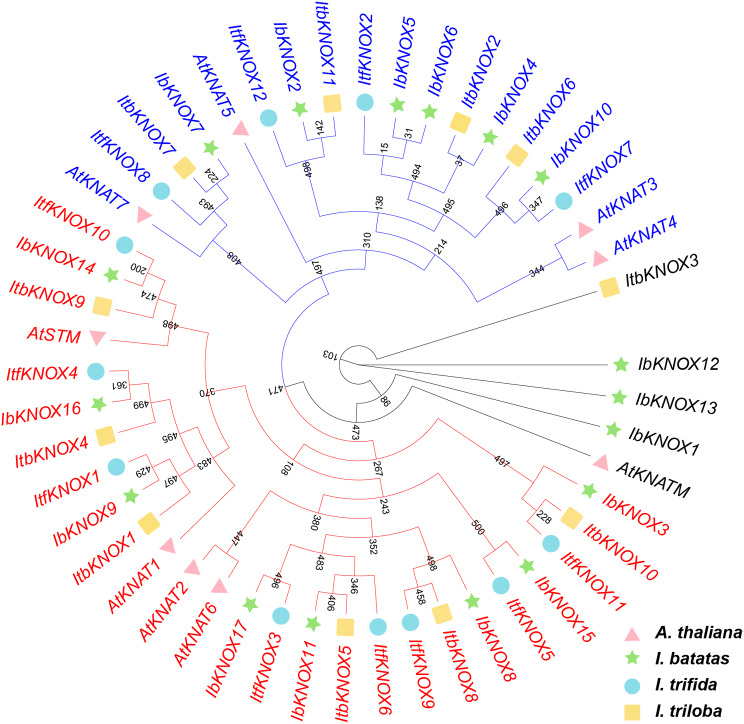
Phylogenetic analysis of the KNOXs in *I. batatas*, *I. trifida*, *I. triloba*, and *A. thaliana.* The green pentagram, blue circles, yellow squares, pink triangles respectively represented the 17 IbKNOXs in *I. batatas*, 12 ItfKNOXs in *I. trifida*, 11 ItbKNOXs in *I. triloba*, and 9 AtKNATs in *Arabidopsis thaliana*. The red line represented the Class I, the dark blue line represented the Class II, and the black line represented the Class M

To investigate the evolutionary relationship of KNOXs in *I. batatas*, *I. trifida*, *I. triloba*, and *Arabidopsis*, a phylogenetic tree for 49 KNOXs of these four species (17 in *I. batatas*, 12 in *I. trifida*, 11 in *I. triloba*, and 9 in *Arabidopsis*) was constructed (Fig. 2). The evolutionary tree was clearly divided into three branches, Class I, Class II, and Class M (Fig. 2). The KNOXs of these four species were distributed in three branches as follows (total: *I. batatas*, *I. trifida*, *I. triloba*, *Arabidopsis*): Class I (8, 8, 6, 4), Class II (6, 4, 4, 4) and Class M (3, 0, 1, 1). AtKNAT2 and AtKNAT 6 in Class I and AtKNAT3, AtKNAT4, AtKNAT5 in Class II have no homologous proteins in sweet potato and its two diploid relatives (Fig. 2). KNOXs in Class M in different plants showed a distant genetic relationship (Fig. 2). Our results revealed that the difference in the number and type of homologous proteins in *Arabidopsis*, sweet potato, *I. trifida* and *I. triloba* was due to species specificity. The discrepancy shown in sweet potato and its two diploid relatives might be attributed to chromosomal hybridization during evolution.

### Conserved domains and exon‒intron structure analysis of *KNOXs* in sweet potato and its two diploid relatives

**Fig. 3:**
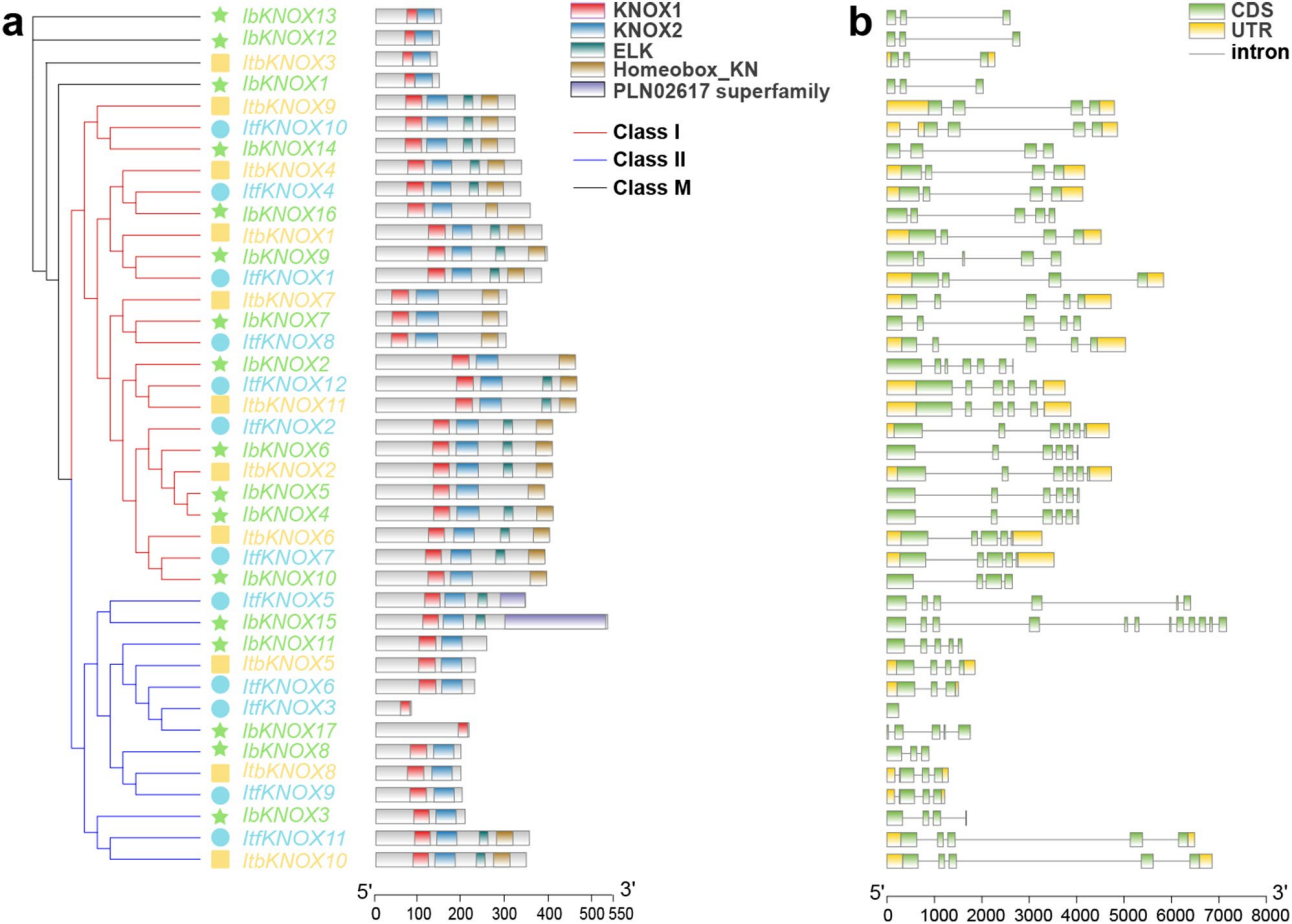
Conserved domains and exon-intron structure of *KNOXs* in *I. batatas*, *I. trifida*, and, *I. triloba.* (**a**) Phylogenetic tree and conserved domain structures of KNOXs. The red box represented the KNOX1 domain. The blue box represented the KNOX2 domain. The green box represented the ELK domain. The brown box represented the Homeobox-KN domain. The purple box represented the PLN02617 domain. (**b**) Exon-intron structures of *KNOXs*. The yellow boxes, green boxes, and black lines represented the UTRs, exons, and introns, respectively

To illustrate the structural characteristics of the 40 KNOX proteins from *I. batatas*, *I. trifida*, and *I. triloba*, motif and domain analyses using the MEME website were performed (Fig. 3). A total of four motifs were identified, including the KNOX1 and KNOX2 domains near the N-terminus, the ELK domain, and the homeobox-KN domain near the C-terminus (Fig. 3a). Overall, the protein structure of this family was relatively conserved, with most members characterized by the presence of four domains. KNOX proteins in Class I contained three or four domains, which were divided into two types. Most KNOXs in Class II contained two domains (KNOX1 and KNOX2), except ItfKNOX5, IbKNOX15, ItfKNOX11 and ItbKNOX10, which contained all four domains, and ItfKNOX3 and IbKNOX17, which contained only the KNOX1 domain. KNOXs in Class M contained KNOX1 and KNOX2 domains, which were similar to most KNOXs in Class II (Fig. 3a). They represented a novel type of KNOX TF that lacked the homeobox domain [55]. An interesting phenomenon was observed where proteins with high genetic relationships might contain different numbers of structural domains, with consistency in two diploids (*I. trifida* and *I. triloba*) but fewer in sweet potato (*I. batatas*). IbKNOX16, IbKNOX2, and IbKNOX10 contained one fewer ELK domain, and IbKNOX3 lacked both the ELK domain and the Homeobox-KN domain compared to their homologous proteins (Fig. 3a). In addition, IbKNOX15 and ItfKNOX5 in Class II contained a new PLN02617 domain. *PLN02617* encoded imidazole glycerophosphate synthase, which was a glutamine aminotransferase in histidine biosynthesis [56]. These findings demonstrated that the presence, number, and distribution of different domains within *KNOX* genes were closely related to their sub-Class and homologous genes. We speculate that the ELK domain might be more susceptible to loss during evolution.

To better understand the gene structure of *KNOXs*, we analyzed the exon‒intron structure of *IbKNOXs* (17), *ItfKNOXs* (12) and *ItbKNOXs* (11) (Fig. 3b). The number of exons in the *KNOX* genes ranged from 1 to 12. *KNOX* genes in Class M contained 3 exons, those in Class I contained 4 to 7 exons, and those in Class II contained 1 to 12 exons. The gene structure of some *IbKNOX* genes differed from that of their homologous genes in *I. trifida* and *I. triloba*. *IbKNOX16* in Class I contained 5 exons, while its homologous genes, *ItfKNOX4* and *ItbKNOX4*, contained only 4 exons. *IbKNOX11* and *IbKNOX17* in Class II contained 5 exons, while their homologs, *ItfKNOX3*, *ItfKNOX6* and *ItbKNOX5*, contained 1, 3 and 4 exons, respectively. *IbKNOX3* in Class II contained 4 exons, while its homologous genes, *ItfKNOX11* and *ItbKNOX10*, contained 5 exons. Taken together, these results indicated that the *KNOX* family might have undergone a lineage-specific differentiation event in the sweet potato genome.

### *Cis*-element analysis in the promoter of *IbKNOXs* in sweet potato

**Fig. 4:**
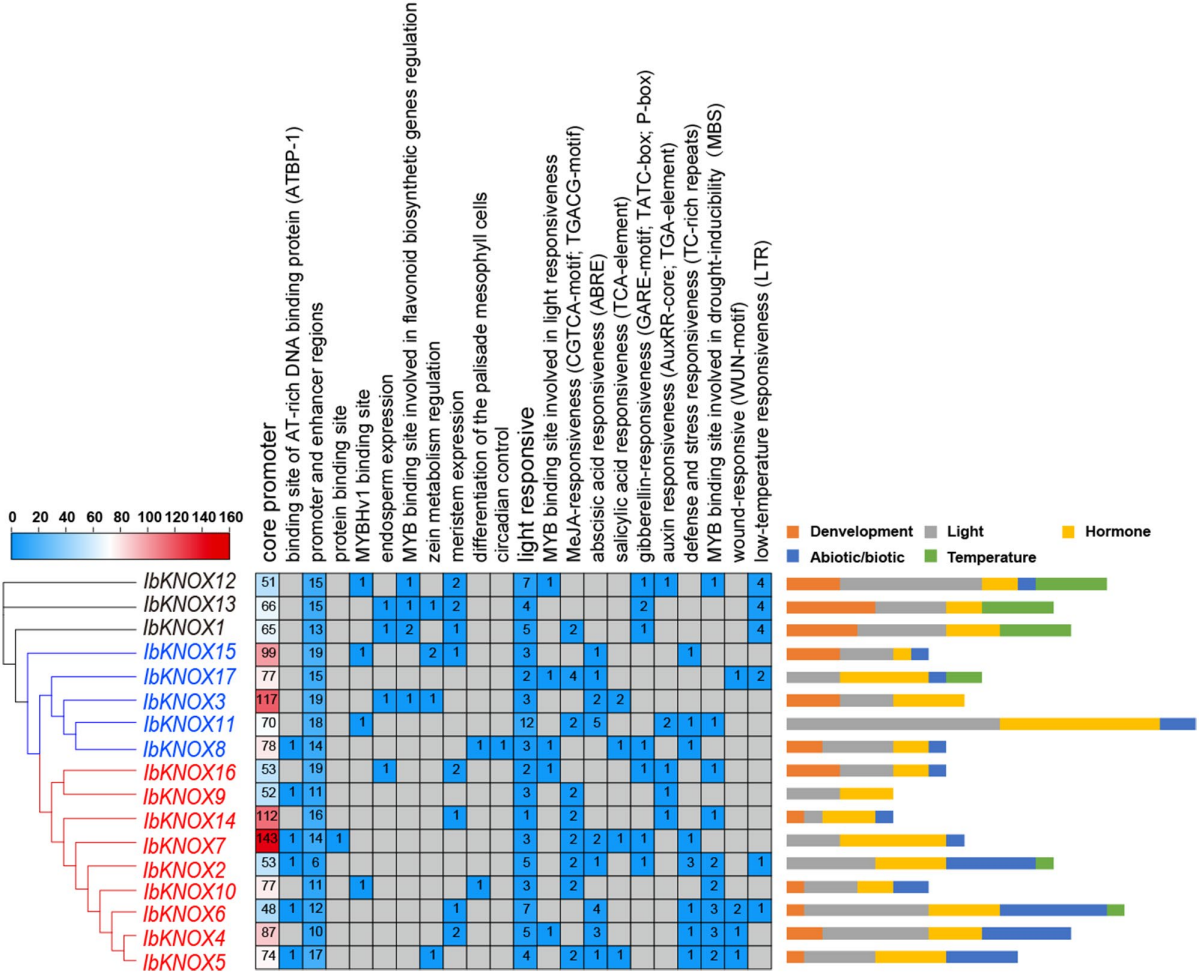
*Cis*-elements analysis of *IbKNOXs* in *I. batatas*. The *cis*-elements were divided into five categories. The depth of different colors represented the number of *cis*-elements in the *IbKNOXs* promoter

Promoter *cis*-elements play a crucial role in initiating gene transcription associated with plant development, hormone regulation, and stress response. To investigate how KNOXs function in growth and development and abiotic stress adaptation in sweet potato, 2000 bp upstream sequences of *IbKNOXs* were extracted, and *cis*-element analysis was performed. According to the functional prediction, the elements were divided into six categories: core/binding sites, development regulation, hormone-responsive, abiotic/biotic stress-responsive, light-responsive and temperature elements (Fig. 4).

All *IbKNOX* genes were found to possess a multitude of core promoter elements, common *cis*-elements, light-responsive elements and some protein binding sites, such as TATA-box, CAAT-box and AT-rich elements (Fig. 4). Development regulation elements were found in most *IbKNOX* genes, such as *cis*-elements related to the meristem, a circadian rhythm control element, an element related to endosperm expression, an element involved in palisade mesophyll cell differentiation and elements involved in zein metabolism (Fig. 4). The hormone-responsive elements in the promoter of *IbKNOXs* were abundant, including MeJA-responsive (CGTCA-motif and TGACG-motif) in *IbKNOX17*, *-11* in Class I, -*9*, *-14*, *-7*, *-2*, *-10*, *-5* in Class II and *− 1* in Class M; ABA-responsive (ABRE) in *-15*, *-17*, *-3*, *-11* in Class I, *-7*, *-2*, *-6*, *-4*, *-5* in Class II; SA-responsive (TCA-element) in *-3*, *-8* in Class I, *-7*, *-5* in Class II; GA-responsive (GARE-motif, TATC-box and P-box) in *-8* in Class I, *-16*, *-7*, *-2* in Class II and *− 12*, *-13*, *-1* in Class M and IAA-responsive (AuxRR-core and TGA-element) in *-11* in Class I, *-16*, *-9*, *-14* in Class II and *− 12* in Class M (Fig. 4). *IbKNOXs* contained three abiotic/biotic stress-responsive elements: defense and stress response element TC-rich repeats, wound-responsive element WUN-motif and MYB binding site involved in drought inducibility MBS (Fig. 4). Overall, *IbKNOXs* might be involved in the regulation of plant growth and development and hormone crosstalk in response to abiotic/biotic stresses in sweet potato through various *cis*-elements in promoters, especially *IbKNOX11* in Class I with the maximum number and *IbKNOX7* in Class II with the maximum type of hormone responsive elements in their promoters.

### Protein interaction network of IbKNOXs in sweet potato

**Fig. 5:**
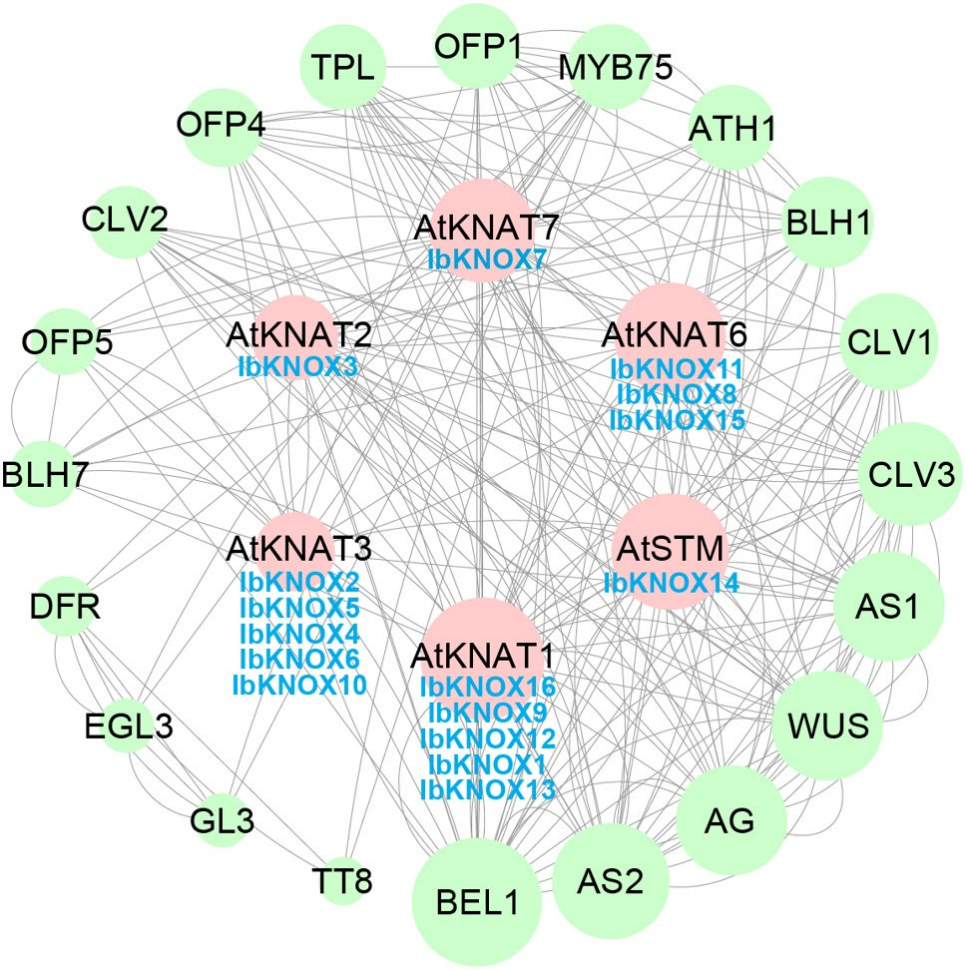
Functional interaction networks of IbKNOXs in *I. batatas* according to orthologues in *Arabidopsis*. Network nodes represented proteins, and lines represented protein-protein associations. The size of the nodes indicated the number of interacting proteins. Dark blue IbKNOXs represented homologous proteins of *Arabidopsis* in *I. batatas*

To explore the potential regulatory network of IbKNOXs, we developed an interaction network based on homologous proteins of *Arabidopsis* (Fig. 5). The results showed that.

IbKNOXs might interact with each other and other proteins, such as floral and vegetative development related protein BEL1 [57], flower development related protein AG (AGAMOUS) [58], MYB transcription factor 75 (MYB75) [59], leaf morphogenesis related protein AS2 (ASYMMETRIC LEAVES 2) [60, 61], organ boundaries development related protein ATH1 (ARABIDOPSIS THALIANA HOMEOBOX GENE1) [62], cell differentiation related protein WUS (WUSCHEL) [63], meristem homeostasis and floral organ numbers regulator CLV3 (CLAVATA3) [64–66], secondary cell wall biosynthesis related proteins OFP1, OFP4 and OFP5 (Ovate Family Proteins) [67, 68] and BEL1-like homeodomain protein BLH1 [68], to regulate ovule and anthocyanin biosynthesis, leaf development and abiotic tolerance (Fig. 5). IbKNOXs interact with ATH1 to form an STM self-activation loop to maintain the self-renewal of the meristem stem cell population. *CLAVATA3* (*CLV3*) and WUSCHEL (WUS) to maintain a constant number of stem cells [64–66]. The MYB75 and OFP4 transcription coregulatory factors could interact with IbKNOX2, -4 ~ 7, and − 10 to regulate the formation of the plant secondary cell wall [69–71]. These results showed that IbKNOXs might be involved in maintaining the state and number of stem cells, regulating hormone biosynthesis and response, and participating in various aspects of plant growth and development.

### Expression analysis of *KNOXs* in sweet potato and its two diploid relatives

#### Expression analysis in various tissues

**Fig. 6:**
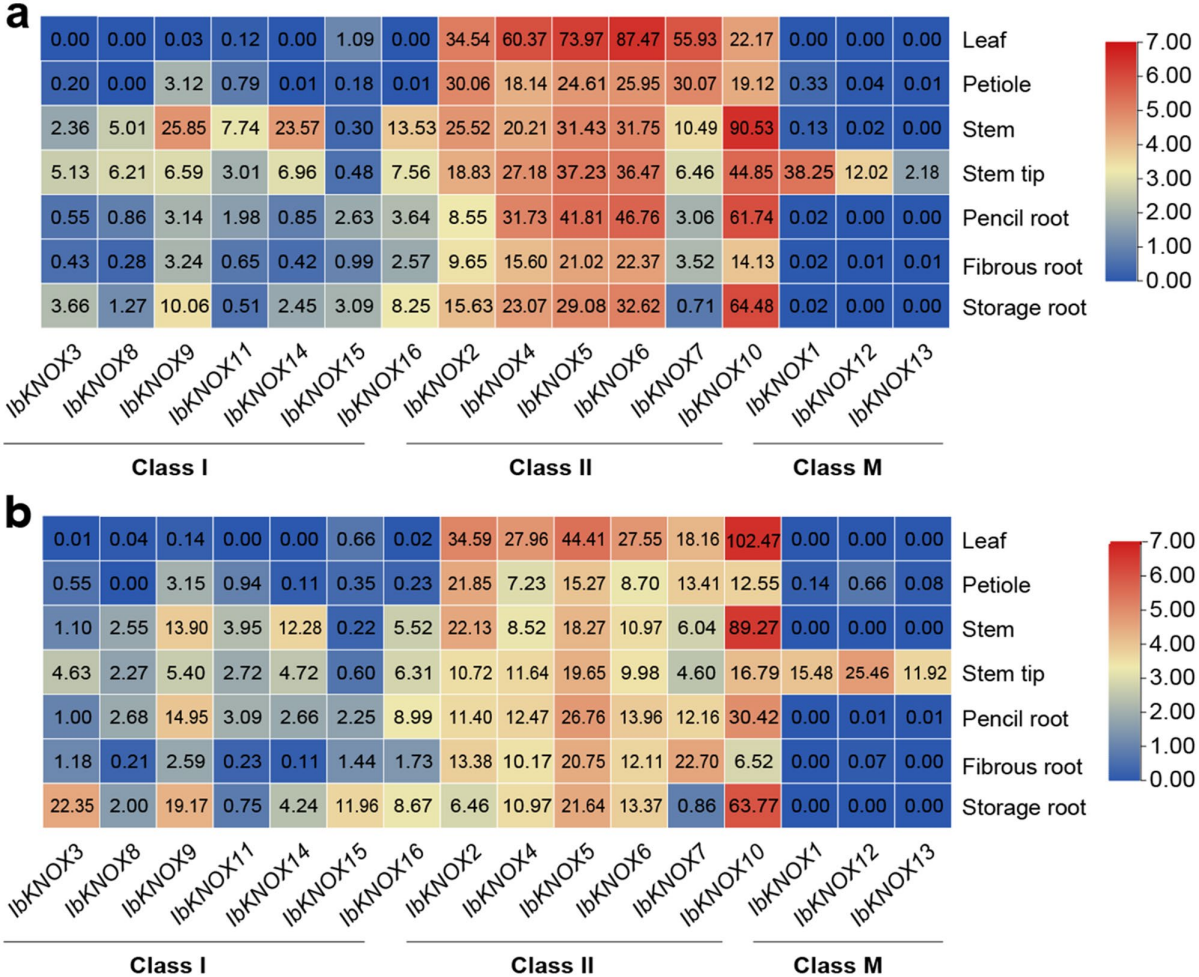
Gene expression patterns of *IbKNOXs* in different tissues. The gene expression patterns of *IbKNOXs* in leaf, petiole, stem, stem tip, pencil root, fibrous root, and storage root of Longshu 9 (**a**) and Xushu 18 (**b**) were determined by RNA-seq. Log2 (FPKM) was shown in the boxes

To explore the potential biological functions of *KNOXs* in the growth and development of sweet potato and its two diploid relatives, we analyzed the expression patterns of *IbKNOXs* in seven tissues (leaves, petiole, stem, stem tip, pencil root, fibrous root, storage root) of Longshu 9 and Xushu 18 (Fig. 6). Longshu 9 and Xushu 18 are varieties with high and stable yields, strong resistance to stress, and wide adaptability [37, 38]. In addition, Longshu9 is precocious [37]. *IbKNOXs* in Class II were widely expressed in various tissues of sweet potato and expressed at higher levels in leaves than in other tissues, while *IbKNOXs* in Class I were more likely to be expressed in stems, stem tips and storage roots, and *IbKNOXs* in Class M were only expressed in stem tips (Fig. 6). The expression patterns of *IbKNOXs* in Longshu 9 and Xushu 18 were similar, except for *IbKNOX9* and *IbKNOX16* in Class I and *IbKNOX7* and *IbKNOX10* in Class II (Fig. 6). IbKNOX9 was highly expressed in stems in Longshu9 (Fig. 6a) but in storage roots in Xushu18 (Fig. 6b). IbKNOX16 was highly expressed in the stem in Longshu9 (Fig. 6a) but in the storage root in Xushu18 (Fig. 6b). IbKNOX7 was highly expressed in leaves in Longshu9 (Fig. 6a) but in fibrous roots in Xushu18 (Fig. 6b). IbKNOX10 leaves were low in Longshu9 (Fig. 6a) and high in Xushu18 (Fig. 6b). These results indicated that *IbKNOX3*, *IbKNOX9*, and *IbKNOX16*, which were highly expressed in storage roots in both Longshu9 and Xushu18, may be involved in the development of storage roots. *IbKNOXs* in Class M may play an important role in plant morphogenesis.

**Fig. 7:**
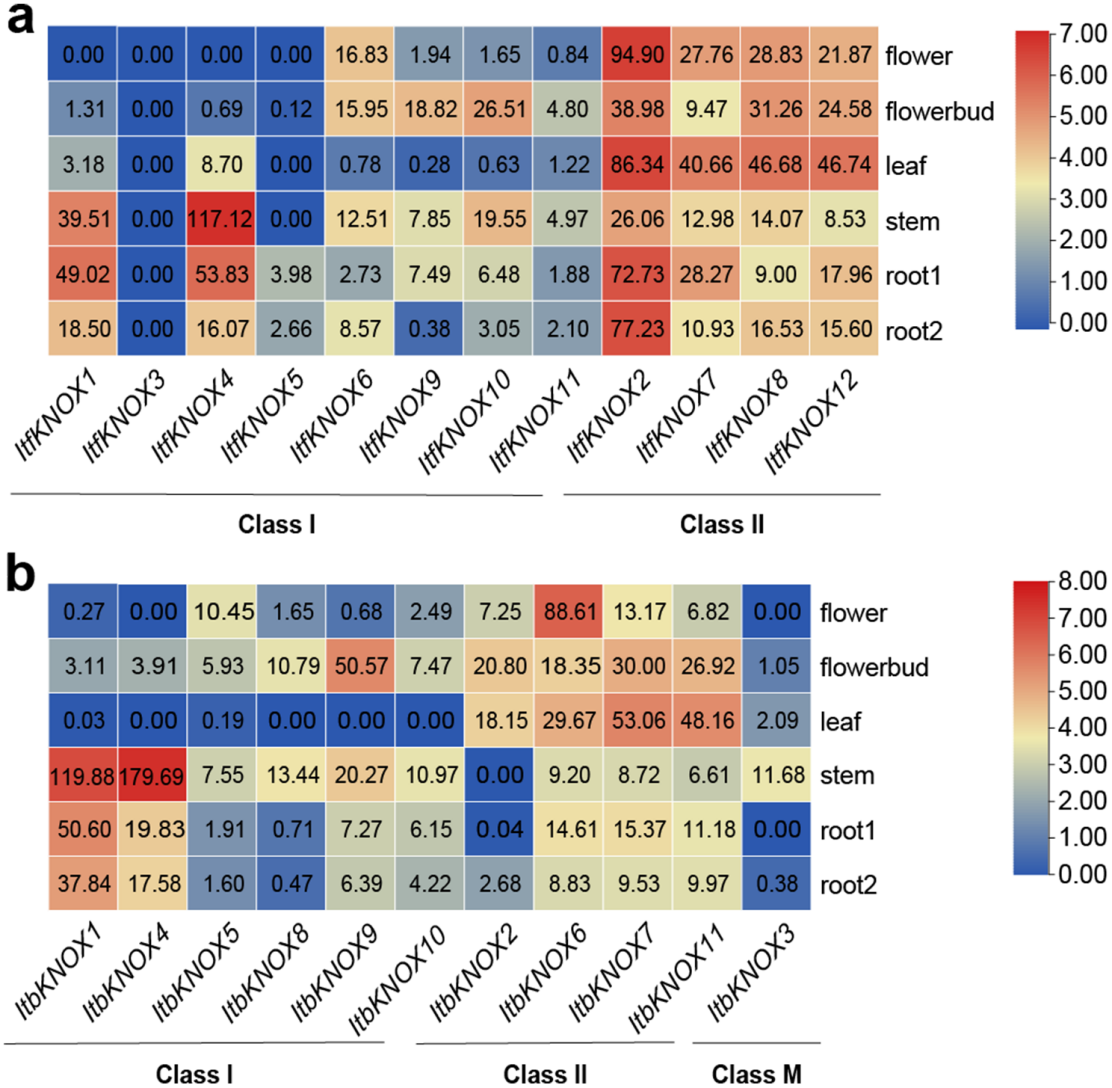
Gene expression patterns of *ItfKNOXs* and *ItbKNOXs* in different tissues. The gene expression patterns of *KNOXs* in flowers, buds, leaf, stem, root 1, root 2 of *I. trifida* (**a**) and *I. triloba* (**b**) were determined by RNA-seq. Log2 (FPKM) was shown in the boxes

The expression patterns of *ItfKNOXs* and *ItbKNOXs* in six tissues (flower, flower bud, leaf, stem, root 1, root 2) of *I. trifida* and *I. triloba* were also analyzed by RNA-seq (Fig. 7). The expression levels of *ItfKNOXs* and *ItbKNOXs* in Class II were significantly higher than those in the other two Classes in all tissues, which was consistent with the results in sweet potato (Fig. 6). In *I. trifida*, *ItfKNOX2* was highly expressed in flowers and flower buds. *ItfKNOX2*, *-7*, *-8* and *− 12* were highly expressed in leaves. *ItfKNOX4* was highly expressed in stems. *ItfKNOX1*, *-2* and *− 4* were highly expressed in root 1, and *ItfKNOX2* was highly expressed in root 2 (Fig. 7a). In *I. triloba*, *ItbKNOX6* was highly expressed in flowers. *ItbKNOX9* was highly expressed in flowerbud. *ItbKNOX7* and *ItbKNOX11* were highly expressed in leaves. *ItbKNOX1* and *− 4* were highly expressed in stems, and *ItbKNOX1* was highly expressed in root 1 and root 2 (Fig. 7b). We found that some homologous genes showed different expression patterns in sweet potato and its two diploid relatives. *IbKNOX10* was highly expressed in the stem and storage root, while its homologous genes *ItfKNOX7* and *ItbKNOX6* were less expressed in the stem and root. The expression levels of *IbKNOX5* and its homologous gene *ItbKNOX2* in roots were low, while the expression levels of *ItfKNOX2* in roots 1 and 2 were high. *IbKNOX9* and *IbKNOX16* were poorly expressed in stems, while their homologous genes were highly expressed in stems. In addition, *IbKNOX9* and *IbKNOX16* were poorly expressed in the storage root, while their homologous genes (except *ItbKNOX4*) were highly expressed in root 1 (Figs. 6 and 7). These results indicated that *KNOXs* had distinct expression patterns in different tissues and that homologous genes in sweet potato and its two diploid relatives were endowed with different functions during evolution.

### Expression analysis of storage roots during different developmental periods of sweet potato

**Fig. 8:**
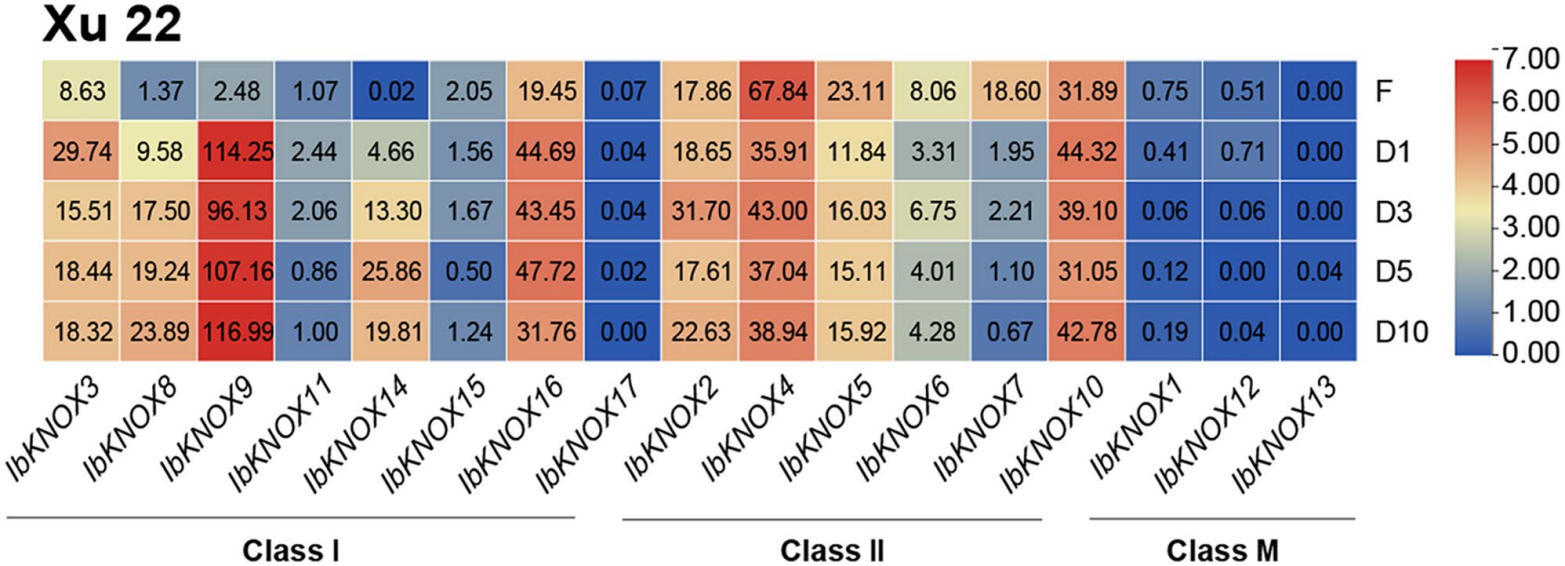
Gene expression patterns of *IbKNOXs* in storage roots in Xu 22 at different periods. F represented fibrous root (diameter of approximately 1 mm), D1 represented initial storage root (diameter of approximately 1 cm), D3 represented storage root (diameter of approximately 3 cm), D5 represented storage root (diameter of approximately 5 cm) and D10 represented storage root (diameter of approximately 10 cm)

Storage root is the main product of sweet potato. The formation of sweet potato storage roots is a complex and changeable process that is related to the downregulation of lignin biosynthesis, upregulation of starch biosynthesis, maintenance of meristem tissue, cell division, and hormonal crosstalk [27, 36]. There was almost no starch accumulation in fibrous roots, while starch accumulated rapidly and continued to increase in the later stage during the early stage of storage root development [36]. To explore the function of *IbKNOXs* in the development of storage roots in sweet potato, we analyzed the expression patterns of *IbKNOXs* in fibrous roots and storage roots with diameters of 1, 3, 5, and 10 cm in the cultivated sweet potato cultivar Xu22 as determined by RNA-seq (Fig. 8, Table S2). *IbKNOX3, -8, -9, -14* and *− 16* in Class I were significantly upregulated in storage roots compared with fibrous roots, among which the expression of *IbKNOX9* increased 46-fold. *IbKNOXs* in Class II, except *IbKNOX2* and *IbKNOX10*, were expressed at higher levels in fibrous roots but at lower levels in storage roots. *IbKNOXs* in Class M were not expressed in either fibrous roots or storage roots (Fig. 8). These results suggested that *IbKNOX2, -3, -8, -9, -10, -14* and *− 16* might be involved in the development of storage roots.

### Expression analysis of hormone response in *I. Trifida* and *I. Triloba*

**Fig. 9:**
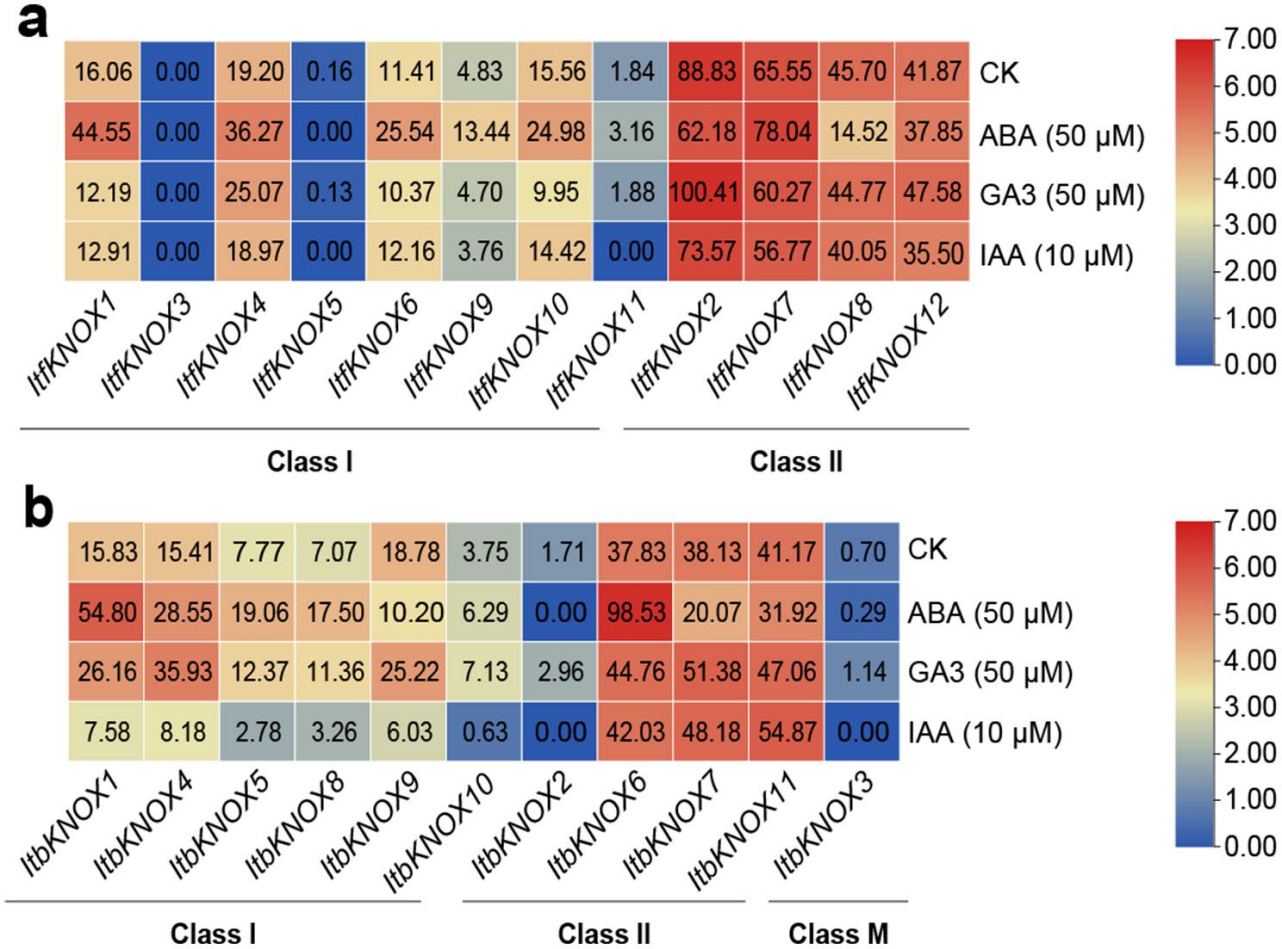
Gene expression patterns of *ItfKNOXs* and *ItbKNOXs* under different hormones treatments. The gene expression patterns of *KNOXs* under ABA, GA3, and IAA treatments in *I. trifida* (**a**) and *I. triloba* (**b**) were determined by RNA-seq. CK: Hormone control. Log2 (FPKM) was shown in the boxes

We analyzed the expression patterns of *ItfKNOXs* and *ItbKNOXs* in *I. trifida* and *I. triloba* with ABA, GA and IAA treatments as determined by RNA-seq (Fig. 9). The expression patterns of homologous genes in *I. trifida* and *I. triloba* were similar. The expression levels of *KNOXs* in Class II were higher than those in Class I with or without treatments. Most *ItfKNOXs* and *ItbKNOXs* were induced by ABA and not very insensitive to GA3 and IAA (Fig. 9). However, *ItfKNOX1* was inhibited, but *ItbKNOX1* was induced by GA3. *ItfKNOX10* was induced by ABA and inhibited by GA3, while its homologous gene *ItbKNOX9* showed the opposite expression pattern. Itf*KNOX2* was highly expressed under the treatment of three hormones in *I. trifida*, while its homologous gene *ItbKNOX2* was almost not expressed in *I. triloba* under treatments. *ItfKNOX8* was inhibited by IAA, but its homologous gene *ItbKNOX7* was induced. Among all the *ItfKNOXs* and *ItbKNOXs*, only *ItbKNOX6* could be induced by all three hormones (Fig. 9). These results showed that the homologous genes of the two diploids had different responses to different hormone treatments, indicating that *ItfKNOXs* and *ItbKNOXs* may be involved in different hormone pathways.

### Expression analysis under abiotic stresses

**Fig. 10:**
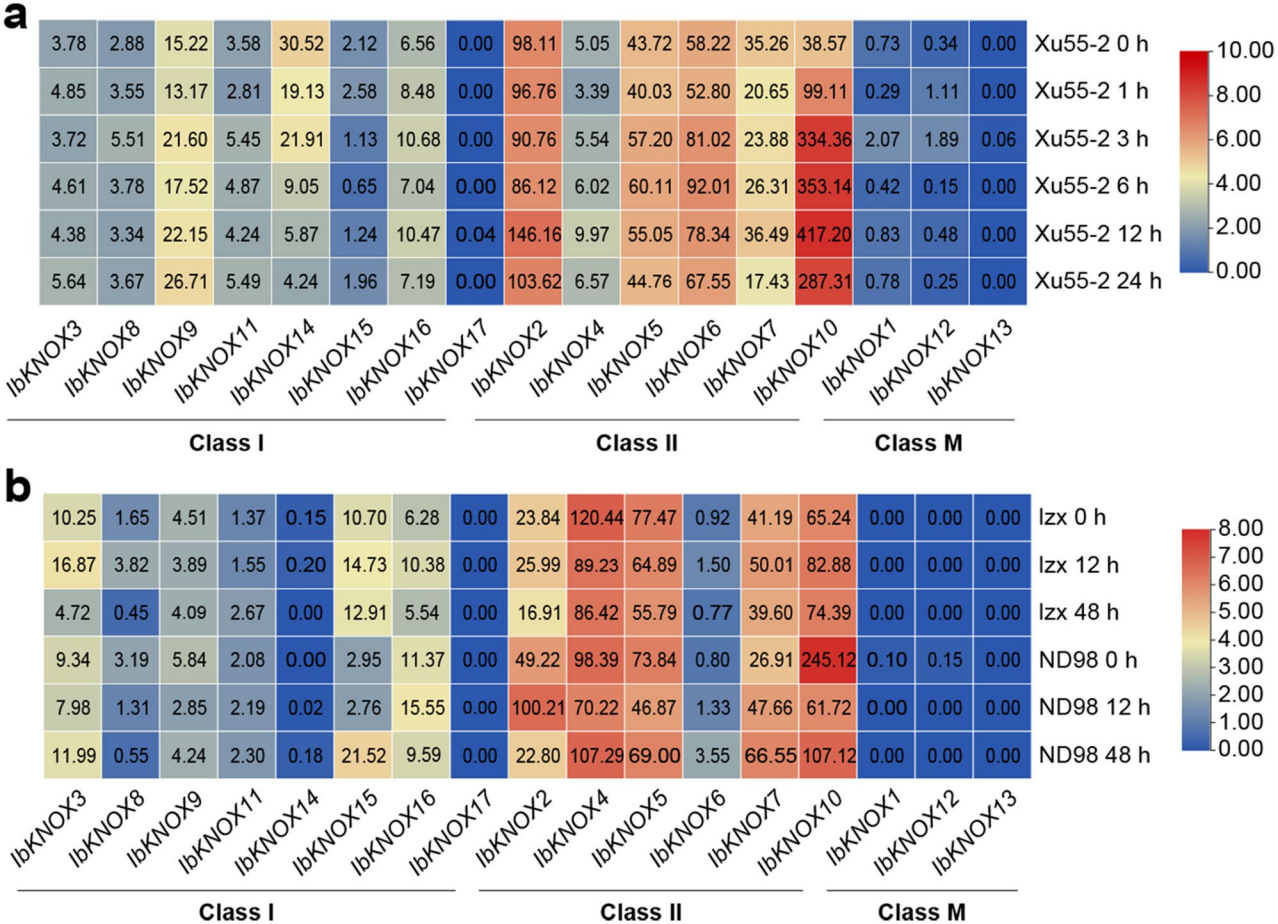
Gene expression patterns of *IbKNOXs* under PEG and NaCl treatments. (**a**) Expression analysis of *IbKNOXs* under PEG treatment in a drought-tolerant line Xu55-2. (**b**) Expression analysis of *IbKNOXs* under NaCl treatment in a salt-sensitive variety lzx and a salt-tolerant line ND98. Gene expression level data were determined by RNA-seq. Log2 (FPKM) was shown in the boxes

To explore the role of *IbKNOXs* in abiotic stresses, the expression patterns of *IbKNOXs* in the drought-tolerant line Xu55-2 under PEG (30%) treatment, salt-sensitive cultivar Lizixiang and salt-tolerant line ND98 under NaCl (200 mM) treatment by RNA-seq were analyzed (Fig. 10, Tables S3 and S4). *IbKNOXs* in Class II showed a significantly higher degree of expression than those in Class I. *IbKNOX9* in Class I and *− 6* and *− 10* in Class II were significantly induced by PEG, especially *IbKNOX10*. However, *IbKNOX14* in Class I and *− 7* in Class II were significantly inhibited by PEG. *IbKNOX1* and *− 12* in Class M were also induced by PEG treatment (Fig. 10a, Table S3). *IbKNOX15* in Class I and *− 2*, *-6*, *-7* in Class II were upregulated by NaCl in ND98 compared with lzx, suggesting that they might be involved in salt stress tolerance. *IbKNOXs* in Class M did not respond to NaCl treatment (Fig. 10b, Table S4). The expression levels of *IbKNOX2* and *IbKNOX6* were induced by PEG and NaCl treatments, which indicated that they might be involved in both drought and salt stress tolerance in sweet potato (Fig. 10).

To prove the expression pattern of *IbKNOXs*, we performed qRT-PCR analysis to verify the expression levels of *IbKNOXs* under NaCl and PEG treatments. The results showed that *IbKNOX2, -4, -6, -10* were upregulated significantly and *− 14*, *-16* were downregulated by PEG treatment (Fig. S1a-f; Table S5). *IbKNOX2, -6, -7* and *− 15* were upregulated significantly by NaCl treatment (Fig. S1g-j; Table S5). *IbKNOX2* and *− 6* were both upregulated by NaCl and PEG (Fig. S1; Table S5), which were consistent with RNA-seq data.

**Fig. 11:**
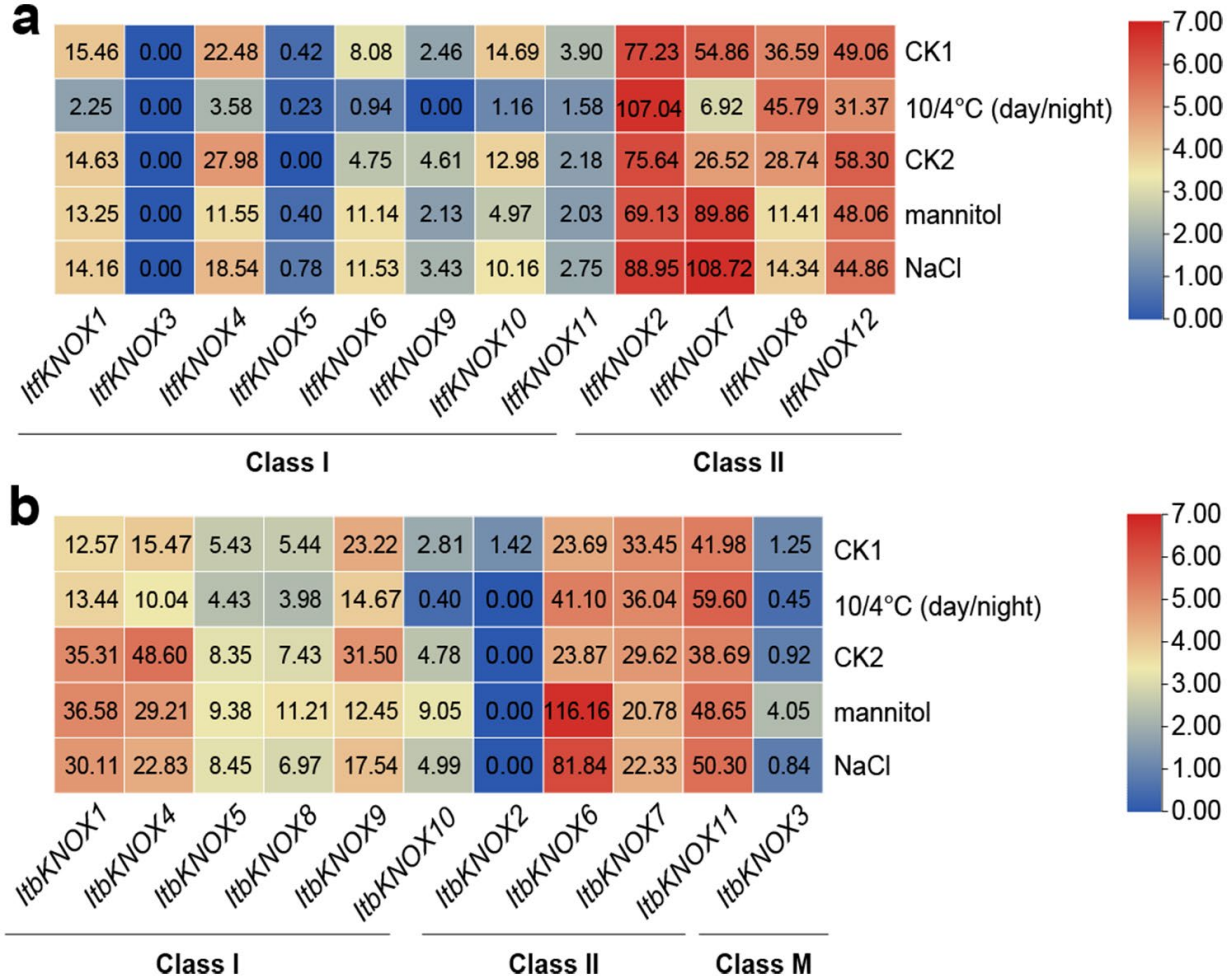
Gene expression patterns of *ItfKNOXs* and *ItbKNOXs* under abiotic stresses. (**a**) Expression analysis of *ItfKNOXs* under 10/4 ℃ (day/night), mannitol and NaCl treatments in *I. trifida*. (**b**) Expression analysis of *ItbKNOXs* under 10/4 ℃ (day/night), mannitol and NaCl treatments in *I. triloba*. CK1: Cold control, CK2: Mannitol and NaCl control. Gene expression level data were determined by RNA-seq. Log2 (FPKM) was shown in the boxes

The expression patterns of *ItfKNOXs* and *ItbKNOXs* in *I. trifida* and *I. triloba* treated with mannitol, NaCl and low temperature (10/4°C day and night) were determined by RNA-seq (Fig. 11). Under low-temperature stress, the expression of *ItfKNOXs* was inhibited, except for *ItfKNOX2* and *− 8* in *I. trifida* (Fig. 11a). In *I. triloba*, the expression levels of *ItbKNOX6* and *− 11* were upregulated, while the expression levels of *ItbKNOX4* and *− 9* were downregulated (Fig. 11b). Under mannitol and NaCl treatments, the expression levels of most homologous *KNOXs* were similar, except *ItfKNOX6/ItbKNOX5*, *ItfKNOX9/ItbKNOX8 and ItfKNOX11/ItbKNOX10*. *ItfKNOX6* was induced, but *ItbKNOX5* did not respond to mannitol and NaCl. *ItfKNOX9* was inhibited, and *ItbKNOX8* was induced. *ItfKNOX11* did not respond to mannitol, but *ItbKNOX10* was induced (Fig. 11b). These results indicate that the expression pattern of this gene has changed in sweet potato and its two diploid relatives.

## Discussion

*KNOX* genes have been reported to be involved in plant growth and development, drought and salt stress, and hormone regulation in a variety of crops [7, 20, 23, 72, 73]. However, the *KNOX* gene family in sweet potato has not been fully analyzed. Sweet potato (*I. batatas*) is an autohexaploid (2n = 6x = 90) varying from *I. trifida* NCNSP0306 (2n = 2x = 30) and *I. triloba* NCNSP0323 (2n = 2x = 30) and is an important crop because of its storage root [33, 74]. Moreover, *I. trifida* showed better stress tolerance [75]. The difference between sweet potato and its two diploid relatives can help to identify the key genes related to storage root development and abiotic tolerance.

The *KNOX* gene family has been reported in many species [5–7, 11–14, 76]. In this study, a total of 40 *KNOX* genes, *I. batatas* (17), *I. trifida* (12) and *I. triloba* (11), were identified (Fig. 1). *KNOXs* in sweet potato contained 5 and 6 more genes than its two diploid relatives, respectively, indicating that *KNOX* genes were amplified in sweet potato compared with its two diploid relatives. Sequence differences between genomes and chromosome differentiation reveal the direction of evolution [77]. The location and distribution of *KNOX* genes on the chromosomes of sweet potato were significantly different from those in its two diploid relatives, while there were only two differences on chromosomes between the two diploid relatives (Fig. 1). According to the phylogenetic relationship with *Arabidopsis thaliana*, KNOXs were divided into three Classes (Class I, Class II, Class M) (Fig. 2). *I. batatas* and *I. triloba* contained 3 *IbKNOXs* and 1 *ItbKNOX* in Class M, respectively, while *I. trifida* did not contain *ItfKNOXs* in Class M (Fig. 2). The exon‒intron distributions of some *IbKNOXs* in *I. batatas* were different from their homologous genes in *I. trifida* and *I. triloba* (Fig. 3b). *IbKNOX16* in Class I contained five exons, while its homologous genes *ItfKNOX4* and *ItbKNOX4* contained four exons (Fig. 3b). *IbKNOX3* in Class II contained three introns, while its homologous genes *ItfKNOX11* and *ItbKNOX10* contained four introns (Fig. 3b). The results indicated that a complex evolutionary process took place in the evolution of sweet potato and its two diploid relatives.

KNOX proteins play important roles in regulating plant organ differentiation [78–80]. In this study, the expression patterns of many *KNOXs* showed tissue specificity (Fig. 6). It is indicated that KNOXs might participate in regulating organ differentiation of sweet potato. The result of KNOX protein interaction network showed that IbKNOXs might interact with BEL1 [57], MYB75 [59] and OFPs [67, 68]. In tomato, SlKN5-SlBLH regulatory modules inhibited fruit greening [81]. In *Arabidopsis thaliana*, both MYB6 and MYB75 interacted with KNAT7 to regulate secondary cell wall formation [59, 82]. OFPs, which often interact with both Class I and II KNOX proteins [83] and also BELL proteins to form OFP/KNOX/BELL complexes [71, 84, 85], control fruit shape and secondary cell wall biosynthesis. It should be noted that Class I KNOX proteins can control secondary cell wall (SCW) and lignin biosynthesis through GA signal pathway [86, 87]. In this study, the promoters of more than one *IbKNOXs* contained GA responsive elements (Fig. 4). It is worth investigating if IbKNOXs interact with BEL/MYB/OFP proteins to regulate SCW and lignin biosynthesis during the development of storage roots in such a pathway.

*KNOXs* are mainly expressed in the root, stem, leaf, flower and shoot tip meristem in dicotyledons and in the stem, meristem and spike in monocotyledons [2, 5, 6, 8–10, 12, 13, 16]. *KNOX* I genes had been reported to be involved in the development of sweet potato storage roots and regulate the level of cytokinin in storage roots [26]. During the development of storage roots, *Ibkn2* (*IbKNOX9* in this study) and *Ibkn3* (*IbKNOX16* in this study) were highly expressed, while *Ibkn1* (*IbKNOX14* in this study) and *Ibkn3* were highly expressed in mature stem internodes [26], and their expression was higher in storage roots than in fibrous roots [27]. In this study, *IbKNOX4, -5*, and *− 6* were highly expressed in the leaves of the high-yield varieties Longshu9 and Xushu18 (Fig. 6), indicating that they might regulate the development of leaves. Interestingly, *IbKNOXs* in Class M were specifically expressed in the stem tip and hardly expressed in other tissues, suggesting that they might play an important role in the development of meristem tissue (Fig. 6). In addition, the expression levels of *IbKNOX14* (*Ibkn1*), *-9* (*Ibkn2*) and *− 16* (*Ibkn3*) in initial storage roots were increased compared to those in fibrous roots (Fig. 8), which was consistent with previous studies. These results indicate that these three genes may be related to the development of storage roots. Moreover, *IbKNOX3* and *IbKNOX8* in Class I were upregulated in initial storage roots compared to fibrous roots (Fig. 8). Notably, the promoters of *IbKNOX14*, *-9*, *-16*, *-3* and *− 8* contained more than one hormone responsive elements, such as ABA, IAA, GA and MeJA (Fig. 4). The development of storage roots in tuberous crops is a complex process, which is regulated by multiple hormone signaling pathways [88, 89]. Based on the above results, we speculated that *IbKNOX14*, *-9*, *-16*, *-3* and *− 8* might be involved in the development of storage roots through ABA, SA and GA signaling pathways.

Abscisic acid (ABA) is a stress resistance hormone in plants. Abiotic stresses, such as salt stress, drought and low temperature, in land plants can increase the endogenous level of ABA [90]. ABA responds to abiotic stress by inducing stomatal closure and root development and promoting ROS clearance, ion transport and osmotic adjustment [91–95]. Accumulating evidence has shown that the increase in endogenous GA3 and IAA levels could promote the expansion and division of leaf epidermal cells [96], and GA3 and IAA are also involved in abiotic stress tolerance [97–100]. In this study, *IbKNOX2*, *-7* and *− 10*, which contained some abiotic and hormone response elements in their promotors, were induced by PEG and NaCl treatments, which indicated that they might be involved in both drought and salt stress tolerances in sweet potato (Figs. 4 and 10). The homologous genes of *IbKNOX2* and *− 10* in two diploid relatives, *ItbKNOX6*, *ItfKNOX7*, and *ItbKNOX11*, were also induced by mannitol and NaCl treatments (Figs. 2 and 11). In *I. trifida, ItfKNOX6* was induced by ABA, mannitol and NaCl, which contained one response element and two low-temperature response elements in the promotor of its homologous gene (Figs. 2, 4, 9a and 11a). *ItfKNOX2* was induced under cold and NaCl treatments and induced by GA3, which contained one ABA response element and two MYB binding sites involved in drought inducibility in the promotor of its homologous gene (Figs. 2, 4, 9a and 11a). These results indicated that these genes might be involved in the response of sweet potato to abiotic stress tolerance through hormone signaling pathways.

## Conclusion

In this study, 17, 12, and 11 *KNOX* genes in sweet potato (*I. batatas*, 2n = 6x = 90) and its two diploid relatives, *I. trifida* (2n = 2x = 30) and *I. triloba* (2n = 2x = 30), were identified. There were differences in protein physicochemical properties, chromosomal localization, phylogenetic relationships, gene structure, protein interaction networks and promoter *cis*-elements among these 40 *KNOX* genes. Their expression patterns in different tissues during different periods of storage root development under different hormones and abiotic stresses, as determined by RNA-seq data, showed tissue specificity and indicated that homologous *KNOXs* might be involved in distinct hormone crosstalk and abiotic stress responses to regulate the growth and development of sweet potato. Among them, *IbKNOX4, -5*, and *− 6* (highly expressed in the leaves), *IbKNOX14*, *-9*, *-16*, *-3* and *− 8* (higher expression in initial storage roots than fibrous roots), and *IbKNOX2* and *− 6* (induced by PEG and NaCl treatments) might be involved in the growth and development of sweet potato storage roots. This study provides a theoretical basis and potential candidate genes for further functional characterization and for improving the yield and abiotic stress tolerance of sweet potato and other species.

### Electronic supplementary material

Below is the link to the electronic supplementary material.

Supplementary Material 1

Supplementary Material 2

## Data Availability

The datasets generated and/or analysed during the current study are available in the NCBI SRA repository (http://www.ncbi.nlm.nih.gov/Traces/sra) under accessions SAMN10755180-SAMN10755194, SRP092215, PRJNA999504, SRP132113, SRP132112, SRP162110, and SRP162021. The datasets unpublished used and/or analyzed during the current study can be obtained from the corresponding author upon reasonable request.
