# Supplementary material for: Genome-wide identification and expression analysis of the KNOX family and its diverse roles in response to growth and abiotic tolerance in sweet potato and its two diploid relatives
Source: BMC Genomics. 2024 Jun 6;25:572. doi: 10.1186/s12864-024-10470-4 (PMC11157901; doi:10.1186/s12864-024-10470-4)
Supplement: Supplementary file 1 — Supplementary Material 1 [file 12864_2024_10470_MOESM1_ESM.docx]

**Table S1** Sequences of the primers used in this study.

| **Primer name** | **Primer sequence (5’-3’)** |
| --- | --- |
| q*β-actin*-F | AGCAGCATGAAGATTAAGGTTGTAGCAC |
| q*β-actin*-R | TGGAAAATTAGAAGCACTTCCTGTGAAC |
| q*IbKNOX2*-F | CCTCGAATCAGTGGCTCTCG |
| q*IbKNOX2*-R | CGATTCGTGCATAATCGCCG |
| q*IbKNOX4*-F | TCTCCATCTCCAGACCACCA |
| q*IbKNOX4*-R | ATTCATCGGCTCCGAAACGG |
| q*IbKNOX6*-F | TCTCCATCTCCAGACCACCA |
| q*IbKNOX6*-R | ATTCATCGGCTCCGAAACGG |
| q*IbKNOX7*-F | TGGATCAATCTGCAGGAGGTG |
| q*IbKNOX7*-R | TCCTGTCGAACCCTCTCCAT |
| q*IbKNOX10*-F | AGGTGAAGGAACAGGTGCAA |
| q*IbKNOX10*-R | AAGCCCATGGTGTCTGCTAC |
| q*IbKNOX14*-F | TGGTGGCGGTTCTATTGCTT |
| q*IbKNOX14*-R | CCTGCGGAACCCTCGTTATT |
| q*IbKNOX15*-F | TGTACGGAGTGAGGTCGTCT |
| q*IbKNOX15*-R | GATCCGGCGGAGAGAAATCC |
| q*IbKNOX16*-F | GCTAGGGGAAGAAGCCGC |
| q*IbKNOX16*-R | CTTGCAAGTGAAACTCGCCG |

**Table S2** Relative expression levels of *IbKNOXs* in storage roots in Xu 22 at different periods.

| **Xu 22** | | **F** | **D1** | **D3** | **D5** | **D10** |
| --- | --- | --- | --- | --- | --- | --- |
| **Class I** | ***IbKNOX3*** | 8.63a | 29.74a | 15.51a | 18.44a | 18.32a |
|  | ***IbKNOX8*** | 1.37c | 9.58bc | 17.50ab | 19.24ab | 23.89a |
|  | ***IbKNOX9*** | 2.48b | 114.25a | 96.13a | 107.16a | 116.99a |
|  | ***IbKNOX11*** | 1.07a | 2.44a | 2.06a | 0.86a | 1.00a |
|  | ***IbKNOX14*** | 0.02c | 4.66c | 13.30b | 25.86a | 19.81ab |
|  | ***IbKNOX15*** | 2.05a | 1.56a | 1.67a | 0.50a | 1.24a |
|  | ***IbKNOX16*** | 19.45a | 44.69a | 43.45a | 47.72a | 31.76a |
|  | ***IbKNOX17*** | 0.07a | 0.04a | 0.04a | 0.02a | 0.00a |
| **Class II** | ***IbKNOX2*** | 17.86a | 18.65a | 31.70a | 17.61a | 22.63a |
|  | ***IbKNOX4*** | 67.84a | 35.91b | 43.00b | 37.04b | 38.94b |
|  | ***IbKNOX5*** | 23.11a | 11.84b | 16.03ab | 15.11b | 15.92ab |
|  | ***IbKNOX6*** | 8.06a | 3.31b | 6.75ab | 4.01b | 4.28b |
|  | ***IbKNOX7*** | 18.6a | 1.95b | 2.21b | 1.10b | 0.67b |
|  | ***IbKNOX10*** | 31.89a | 44.32a | 39.10a | 31.05a | 42.78a |
| **Class M** | ***IbKNOX1*** | 0.75a | 0.41ab | 0.06b | 0.12b | 0.19ab |
|  | ***IbKNOX12*** | 0.51a | 0.71a | 0.06a | 0.00a | 0.04a |
|  | ***IbKNOX13*** | 0.00a | 0.00a | 0.00a | 0.04a | 0.00a |

Data were presented as means ± SD (*n* = 2). Different lowercase letters indicate significant differences (*P* < 0.05; one-way ANOVA).

**Table S3** Relative expression levels of *IbKNOXs* under PEG treatment in a drought-tolerant variety Xu55-2.

| **Xu 55-2** | | **0 h** | **1 h** | **3 h** | **6 h** | **12 h** | **24 h** |
| --- | --- | --- | --- | --- | --- | --- | --- |
| **Class I** | ***IbKNOX3*** | 3.78ab | 4.85ab | 3.72b | 4.61ab | 4.38ab | 5.64a |
|  | ***IbKNOX8*** | 2.88a | 3.55a | 5.51a | 3.78a | 3.34a | 3.67a |
|  | ***IbKNOX9*** | 15.22c | 13.17c | 21.60ab | 17.52bc | 22.15ab | 26.71a |
|  | ***IbKNOX11*** | 3.58bc | 2.81c | 5.45a | 4.87ab | 4.24abc | 5.49a |
|  | ***IbKNOX14*** | 30.52a | 19.13b | 21.91b | 9.05c | 5.87c | 4.24c |
|  | ***IbKNOX15*** | 2.12a | 2.58a | 1.13a | 0.65a | 1.24a | 1.96a |
|  | ***IbKNOX16*** | 6.56b | 8.48ab | 10.68a | 7.04b | 10.47a | 7.19b |
|  | ***IbKNOX17*** | 0.00a | 0.00a | 0.00a | 0.00a | 0.04a | 0.00a |
| **Class II** | ***IbKNOX2*** | 98.11b | 96.76b | 90.76b | 86.12b | 146.16a | 103.62b |
|  | ***IbKNOX4*** | 5.05ab | 3.39b | 5.54ab | 6.02ab | 9.97a | 6.57ab |
|  | ***IbKNOX5*** | 43.72cd | 40.03d | 57.20ab | 60.11a | 55.05abc | 44.76bcd |
|  | ***IbKNOX6*** | 58.22bc | 52.80c | 81.02ab | 92.01a | 78.34abc | 67.55abc |
|  | ***IbKNOX7*** | 35.26a | 20.65cd | 23.88bc | 26.31b | 36.49a | 17.43d |
|  | ***IbKNOX10*** | 38.57b | 99.11b | 334.36a | 353.14a | 417.20a | 287.32a |
| **Class M** | ***IbKNOX1*** | 0.73b | 0.29b | 2.07a | 0.42b | 0.83b | 0.78b |
|  | ***IbKNOX12*** | 0.34b | 1.11ab | 1.89a | 0.15b | 0.48b | 0.25b |
|  | ***IbKNOX13*** | 0.00a | 0.00a | 0.06a | 0.00a | 0.00a | 0.00a |

Data were presented as means ± SD (*n* = 2). Different lowercase letters indicate significant differences (*P* < 0.05; one-way ANOVA).

**Table S4** Relative expression levels of *IbKNOXs* under NaCl treatment in a salt-sensitive variety Lizixiang and a salt-tolerant line ND 98.

|  | | **lzx 0 h** | **lzx 12 h** | **lzx 48 h** | **ND98 0 h** | **ND98 12 h** | **ND98 48 h** |
| --- | --- | --- | --- | --- | --- | --- | --- |
| **Class I** | ***IbKNOX3*** | 10.25b | 16.87a | 4.72b | 9.34a | 7.98a | 11.99a |
|  | ***IbKNOX8*** | 1.65ab | 3.82a | 0.45b | 3.19a | 1.31b | 0.55b |
|  | ***IbKNOX9*** | 4.51a | 3.89a | 4.09a | 5.84a | 2.85b | 4.24ab |
|  | ***IbKNOX11*** | 1.37a | 1.55a | 2.67a | 2.08a | 2.19a | 2.30a |
|  | ***IbKNOX14*** | 0.15a | 0.20a | 0.00a | 0.00a | 0.02a | 0.18a |
|  | ***IbKNOX15*** | 10.70a | 14.73a | 12.91a | 2.95b | 2.76b | 21.52a |
|  | ***IbKNOX16*** | 6.28ab | 10.38a | 5.54b | 11.37b | 15.55a | 9.59b |
|  | ***IbKNOX17*** | 0.00a | 0.00a | 0.00a | 0.00a | 0.00a | 0.00a |
| **Class II** | ***IbKNOX2*** | 23.84a | 25.99a | 16.91a | 49.22b | 100.21a | 22.80b |
|  | ***IbKNOX4*** | 120.44a | 89.23a | 86.42a | 98.39a | 70.22b | 107.29a |
|  | ***IbKNOX5*** | 77.47a | 64.89a | 55.79a | 73.84a | 46.87a | 69.00a |
|  | ***IbKNOX6*** | 0.92a | 1.50a | 0.77a | 0.80b | 1.33b | 3.55a |
|  | ***IbKNOX7*** | 41.19a | 50.01a | 39.60a | 26.91c | 47.66b | 66.55a |
|  | ***IbKNOX10*** | 65.24a | 82.88a | 74.39a | 245.12a | 61.72b | 107.12b |
| **Class M** | ***IbKNOX1*** | 0.00a | 0.00a | 0.00a | 0.10a | 0.00a | 0.00a |
|  | ***IbKNOX12*** | 0.00a | 0.00a | 0.00a | 0.15a | 0.00a | 0.00a |
|  | ***IbKNOX13*** | 0.00a | 0.00a | 0.00a | 0.00a | 0.00a | 0.00a |

Data were presented as means ± SD (*n* = 2). Different lowercase letters indicate significant differences (*P* < 0.05; one-way ANOVA).

**Table S5** Relative expression levels of *IbKNOXs* under PEG and NaCl treatments by qRT-PCR analysis.

|  | | **0 h** | **1 h** | **6 h** | **12 h** | **24 h** |
| --- | --- | --- | --- | --- | --- | --- |
| **20% PEG6000** | ***IbKNOX2*** | 0.98b | 0.28c | 1.42b | 2.88a | 0.08c |
|  | ***IbKNOX4*** | 1.00b | 0.10c | 1.19b | 4.52a | 0.07c |
|  | ***IbKNOX6*** | 1.00bc | 0.38d | 1.34b | 4.03a | 0.64cd |
|  | ***IbKNOX10*** | 1.00cd | 0.57d | 1.79b | 7.86a | 1.24bc |
|  | ***IbKNOX14*** | 1.00a | 1.14b | 0.23c | 0.96a | 0.18c |
|  | ***IbKNOX16*** | 1.00b | 0.44c | 0.35cd | 1.33a | 0.25d |
| **200 mM NaCl** | ***IbKNOX2*** | 1.00c | 55.81ab | 44.13b | 58.02a | 0.13c |
|  | ***IbKNOX6*** | 1.00c | 9.81c | 38.09b | 75.39a | 0.43c |
|  | ***IbKNOX7*** | 1.00c | 6.82c | 20.23b | 102.41a | 0.11c |
|  | ***IbKNOX15*** | 1.00c | 81.26b | 14.65c | 136.28a | 0.27c |

Data were presented as means ± SD (*n* = 3). Different lowercase letters indicate significant differences (*P* < 0.05; one-way ANOVA).
