# Supplementary material for: Genome-wide identification and expression analysis of the KNOX family and its diverse roles in response to growth and abiotic tolerance in sweet potato and its two diploid relatives
Source: BMC Genomics. 2024 Jun 6;25:572. doi: 10.1186/s12864-024-10470-4 (PMC11157901; doi:10.1186/s12864-024-10470-4)
Supplement: Supplementary file 2 — Supplementary Material 2 [file 12864_2024_10470_MOESM2_ESM.docx]

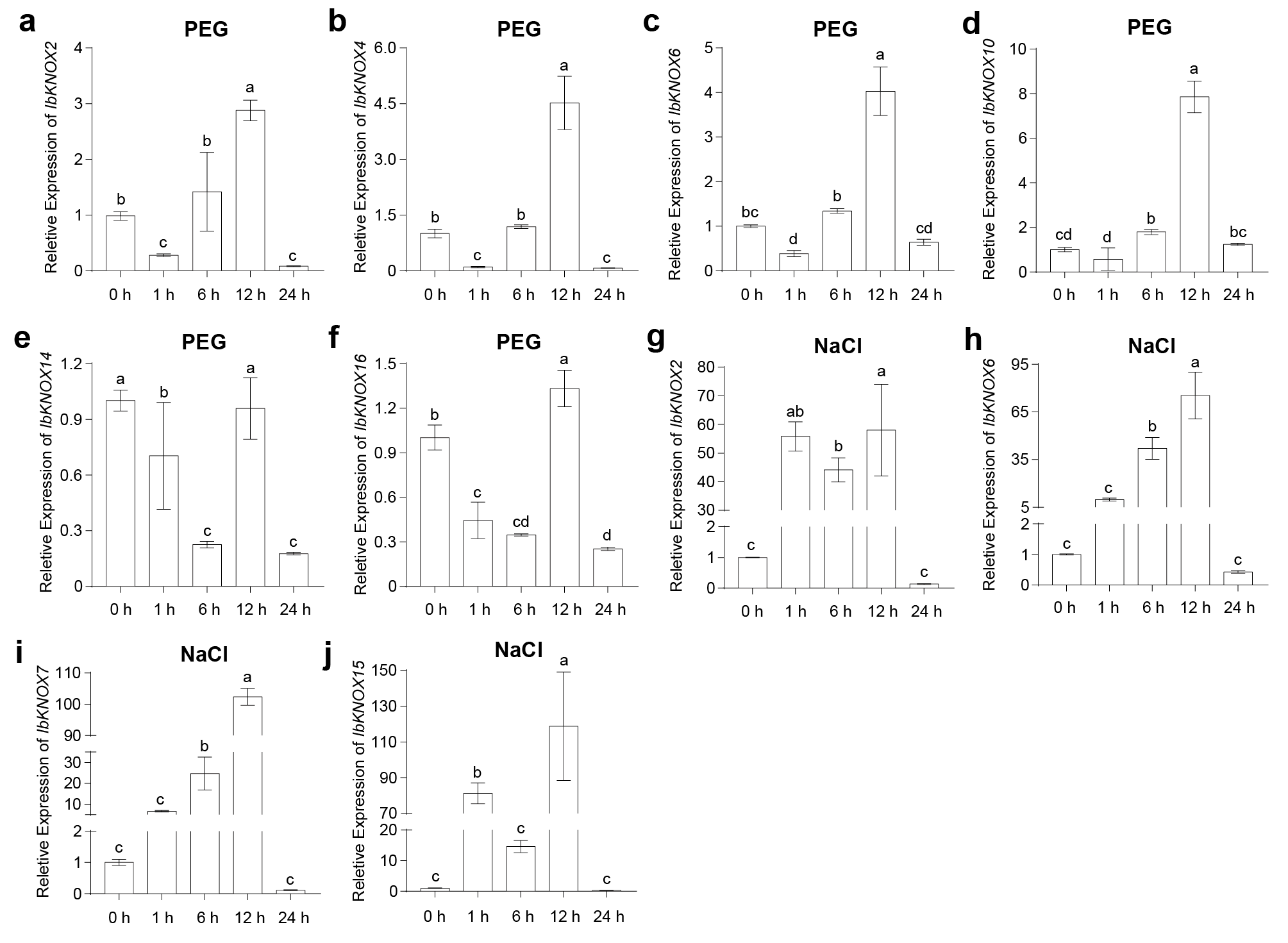


**Fig S1**. The expression levels of *IbKNOXs* under NaCl and PEG treatments by qRT-PCR analysis. (**a-f**) *IbKNOX2*, *-4*, *-6*, *-10*, *-14*, and *-16* were measured in ‘X18’ treated with 20% PEG6000. (**g-j**) *IbKNOX2*, *-6*, *-7*, and *-15* were examined in ‘ND98’ plants treated with 200 mM NaCl. Data are presented as the means ± SD (*n* = 3). Different lowercase letters indicate significant differences (*P* < 0.05; one-way ANOVA).
